# A randomised controlled feasibility study of the Carers-ID intervention to support the mental health of family carers of people with intellectual disabilities

**DOI:** 10.1371/journal.pone.0345096

**Published:** 2026-03-20

**Authors:** Rachel A. Leonard, Maria Truesdale, Michael Brown, Lynne Marsh, Stuart Todd, Nathan Hughes, Trisha Forbes, Ashleen Crowe, Mark A. Linden

**Affiliations:** 1 School of Nursing and Midwifery, Queen’s University Belfast, Belfast, Northern Ireland; 2 School of Health and Wellbeing, University of Glasgow, Glasgow, Scotland; 3 School of Care Sciences, University of South Wales, Caerleon, Wales; 4 Department of Sociological Studies, University of Sheffield, Sheffield, England; University Hospital Cologne: Uniklinik Koln, GERMANY

## Abstract

**Background:**

Intellectual disability (ID) refers to significant limitations in intellectual and adaptive functioning beginning in childhood. Globally, ID affects 1–3% of the population—over 200 million people. Family carers of individuals with ID experience high levels of stress, poor health, and reduced quality of life due to ongoing caregiving demands. These pressures intensified during the COVID-19 pandemic, prompting the development of Carers-ID, an online intervention designed to support carers’ mental health.

**Objective:**

To assess the feasibility of delivering the Carers-ID programme to family carers of people with ID.

**Methods:**

A parallel randomised controlled trial was conducted to evaluate recruitment and retention rates, feasibility of data collection, and potential effect sizes. Carers were recruited via UK-based voluntary organisations and NHS learning disability teams and randomly assigned to either the Carers-ID intervention (n = 51) or waitlist control (n = 48). Randomisation was conducted by an independent third party. The intervention spanned two weeks with assessments at baseline, post-intervention, and three-month follow-up. Outcomes included measures of well-being, resilience, social connectedness, depression, anxiety, and stress. The trial followed CONSORT reporting guidelines.

**Results:**

Of 150 carers screened, 99 met inclusion criteria, and 84 completed the baseline assessment (85%). Retention was 55% post-intervention and 41% at three-month follow-up. Adjusted mean differences between-groups at T2 (2 weeks from baseline) across the four measures were as follows: 3.42 (SE = 2.84, p = 0.23) for wellbeing, 12.20 (SE = 5.83, p = 0.04) for resilience, 5.09 (SE = 5.12, p = 0.32) for social connectedness, 0.98 (SE = 1.40, p = 0.49) for depression, 0.42 (SE = 1.26, p = 0.74) for stress, and 1.06 (SE = 1.32, p = 0.43) for anxiety.

**Conclusion:**

Family carers face time and resource pressures which may exclude them from clinical trials. Challenges in retaining carers highlight the need for flexible intervention formats. Despite retention issues, results suggest feasibility in delivering the Carers-ID intervention. Future effectiveness trials should address barriers to participation and tailor interventions for this underserved population. **Trial registration ClinicalTrials.gov:** NCT05737823

## 1. Introduction

Intellectual disability (ID) refers to a significant impairment of general intellectual and adaptive functioning that originates in childhood [1]. The global prevalence of ID has been estimated at between 1–3% equating to over 200 million people [2]. Family carers of people with intellectual disabilities often play a central and multifaceted role in supporting the health and well-being of their family member. Their caring responsibilities often encompass medication management, physical care, behaviour support, advocacy, and coordination with health and social care services [3]. In England, approximately 77% of people with intellectual disabilities lived with their families in 2017–2018, underscoring the scale and importance of this unpaid caregiving workforce [4]. Despite their critical role, many family carers experience substantial and persistent stress, alongside increased risks of mental health difficulties [5,6].

Interventions that provide structured training and support for family carers have demonstrated positive outcomes, including reductions in stress, improved confidence [7], enhanced child socialisation [8], and overall improvements in quality of life [9]. However, access to these programmes is often limited by practical barriers such as time constraints, financial costs, and availability of services [10]. Online interventions offer a promising alternative by increasing flexibility, accessibility and have shown comparable effectiveness to traditional face-to-face approaches in improving parenting practices, knowledge, and self-efficacy [11,12]. Nevertheless, a systematic review revealed that few online interventions have been co-developed with carers themselves, limiting their relevance and responsiveness to carers’ lived experiences and evolving needs [13].

The COVID-19 pandemic further disrupted access to face-to-face care and support, leading many services to transition to online delivery [14,15]. While this shift introduced new challenges—such as digital literacy and access to reliable technology—it also highlighted potential advantages including reduced travel, lower delivery costs, and increased access to support programmes [16].

Previous trials have tested the feasibility of interventions for carers of those with ID [17–19]. However, these have been tested in specific groups, such as parents of children under 5 years [18], or have tested specific interventions, such as mindfulness [19]. Only one of these interventions have been offered online [19] with the other two being delivered face to face [17,18]. In addition, these have been conducted in small samples, seven parent carers, 60 family carers and 74 family carers respectively [17–19], limiting generalisability to the wider population.

The Carers-ID programme was developed in 2021 (18; Ref: ES/W001829/1) and co-produced with family carers and experts in ID with a primary focus on supporting family carers mental health and well-being. The programme was found to be highly acceptable to family carers across the United Kingdom (UK) [20], however, the feasibility of conducting a larger effectiveness trial was unknown. Therefore, this current study further contributes to our understanding of the feasibility of conducted a larger effectiveness trial. We conducted this study to address whether a randomised controlled trial was feasible to deliver the Carers-ID programme to family carers of people with ID.

### 1.1. Objectives

The aim of this RCT was to assess the feasibility of delivering the Carers-ID programme to family carers of people with ID. The study will inform a potential, definitive RCT of the effectiveness of Carers-ID. The primary objectives were to determine:

1. Recruitment, retention and attrition rates of participation in the trial;
2. Intervention engagement and adherence;
3. Potential effect sizes and variability to inform a sample size calculation in an effectiveness trial;
4. Feasibility of collecting outcome data using measures of stress, anxiety, depression, resilience and wellbeing in assessing the impact of the intervention on family carers.

In addition, a secondary objective was to:

5. Examine the impact on family carers mental health outcomes (stress, anxiety, depression, well-being and resilience) to guide a future trial.

## 2. Materials and methods

### 2.1. Study design

We conducted a parallel-group randomised wait-list controlled trial, with 1:1 randomisation. Although randomisation is not required for feasibility, the chosen design incorporates randomisation not to test effectiveness, but to assess the feasibility of conducting a future definitive RCT. A wait-list control design was employed for ethical reasons, as it allows all participating carers to ultimately receive the intervention. The trial protocol has been published elsewhere [21] and has been registered with the US (United States) National Library of Medicine, Clinical Trials Register (ID: NCT05737823). Following commencement of the study, some changes were made to the original protocol, specifically trialling a new recruitment source for family carers (see 2.4 recruitment via UK National Health Service (NHS) below), and a change in outcome measure timing (see 2.7 outcomes). Ethical approval was received from a Queen’s University Belfast Ethics Review Board (MHLS23_04) and the North-West – Preston Research Ethics Committee (24/NW/0095).

### 2.2. Participants

Participants comprised family carers of people with ID, and were recruited employing two recruitment approaches: (1) carer and disability voluntary sector charities and (2) NHS learning disability teams, across Northern Ireland, Scotland, Wales and England, between May 2023 and January 2025. Eligibility criteria included UK adults > 18 years of age providing care for a family member with an ID (people with all levels of ID).

### 2.3. Recruitment via charities

At the outset of this study, we partnered with voluntary sector charities (Carers UK, ARC Northern Ireland (NI) and All Wales Forum) to facilitate recruitment of family carers. While these charities were the main avenues for recruitment, we also contacted many other charities across the UK to target a wide range of family carers. Charities advertised the study to members via several avenues, including social media, monthly newsletters, and individual emails to members. Potential participants registered their interest by email. This was followed up with a screening email, along with the participant information sheet. Once participants confirmed participation and eligibility, a link to the baseline survey was sent, which contained a consent form.

Rates of recruitment were not as expected therefore an alternative source of recruitment was trialled. In June 2024 recruitment via NHS disability teams in England, Scotland and Wales commenced. Recruitment also continued through the charities; therefore, the two recruitment methods ran concurrently from June 2024 to January 2025.

### 2.4. Recruitment via NHS

Recruitment of family carers began in NHS Community Learning Disability Teams with three health service areas across the UK (NHS Lothian, North Staffordshire Combined Healthcare NHS Trust, and Swansea Bay University Health Board). Ethical approval also commenced with a Trust in Northern Ireland, however, due to delays in this process, recruitment of family carers within the time frame of the study was not possible. Local collaborators were recruited to act as gatekeepers for the Community Learning Disability Teams to facilitate recruitment. The Community Learning Disability Teams identified eligible family carers and distributed study information via different avenues, including, post, email and visiting eligible carers. Teams also advertised the study recruitment flyer in clinics and social media. Potential participants registered their interest by email and the same procedure outlined above was followed.

### 2.5. Process evaluation

Participants who completed the intervention were invited to participate in an online interview. An invitation letter and information sheet were sent to all participants in the intervention group via email. Participants registered their interest and a suitable time and date was agreed. All interviews were conducted online via Microsoft Teams by the first author (RL), with informed consent taken prior to commencement. Interview questions focused on their overall experience, relevance, usefulness, and potential impact of the intervention. For example, ‘What improvements do you think could be made to the Carers-ID programme?’ and ‘What has been the impact of the Carers-ID online programme on you and/or your family?’

### 2.6. Intervention description and procedure

Carers-ID was designed and co-produced with family carers and ID experts with a primary focus on supporting well-being. The intervention (www.Carers-ID.com) is delivered online and comprises video and audio accounts of family carers’ experiences, peer-to-peer support, resources and activities which promote resilience and improve mental health. Carers-ID consists of 14 modules which cover topics comprised the following: promoting resilience, providing peer support, reducing anxiety, managing stress, accessing local supports and managing family conflict and information for siblings who are carers. It also shares examples of carers’ experiences of the COVID-19 pandemic and offers day-today accounts of successful strategies individuals used to improve their mental health. The Carers-ID website is openly accessible; however, some modules were restricted. Five modules were freely available, however, to access the remaining 9 modules participants required a log in name and password. Therefore, once those in the intervention group had completed their baseline measures, the research team provided them with access details (username and password) and instructions on how to navigate the website. Access was not time limited; those with log in details (including those in the control group who had completed the study) would have access to the intervention on a permanent basis. The online programme was asynchronous and self-directed by participants. Participants were requested to complete the intervention within a two-week period, there was no set number of hours required to complete the intervention.

The Carers-ID intervention also comprised monthly peer support sessions, facilitated by family carers. These sessions connected family carers with each other so they could provide peer support and mentoring. Facilitators were recruited on a rolling basis from the beginning of the study. The research team contacted enrolled participants within the intervention group, and those in the control group who had completed the 2^nd^ measure, to offer the opportunity to facilitate sessions. Facilitators were provided with guidelines on how to manage sessions including how to spot and manage signs of distress. Times and dates were arranged between facilitators and the research team (a range of times and dates were used to suits carers, i.e., during the day, evening) and advertised to all participants within the intervention group, and those in the control group who had completed the 2^nd^ measure time point (T1). Sessions were voluntary and were not mandatory as part of the intervention. Sessions were delivered online (via zoom) and lasted approximately an hour. Sessions were informal and did not follow a set structure or theme. The facilitator typically started off the conversation and followed the thread of what others wanted to discuss. A member of the research team was always present at these sessions to monitor technical issues and ethical issues, such as safety of participants.

Participants in the waitlist control group received no active intervention. These individuals gained access to the intervention following completion of the 2^nd^ measure (T1), which was the end point of the study for the control group.

### 2.7. Outcomes

The main outcomes were feasibility of delivery of the intervention to participants, with pre-defined criteria for determining progression to a largescale effectiveness trial (Table 1). These criteria included:

**Table 1:** Comparison of the results with criteria for progression.

	Criteria for study success	Results
**Feasibility**		
***Recruitment***	Recruitment of > 90 family carers	99 family carers were successfully recruited
***Retention***	Retention rates of > 80% family carers	64 participants were retained at 2 weeks (65% retention rate)
***Outcome measure feasibility***	Measures completed by > 80% of family carers	64 participants completed the 2 week measure (65%)
***Outcome measure acceptability***	Relevance and usefulness of outcome measures	“didn’t find them intrusive” “they're getting you to think about how you're feeling”

1. Acceptability and feasibility of the outcome measures (completed by > 80% of family carers)
2. Sufficient recruitment (> 90 carers) and participation and retention rates (> 80% of family carers)
3. Effect sizes and estimate of variability (standard error), along with data from previous studies, to inform sample size for a future effectiveness trial.

To assess the primary objective of recruitment, retention and attrition rates of participation in the trial we collected data on numbers of participants identified, recruited, commenced and who completed the study. Participants who declined participation or those that dropped out were followed up to determine their reasons for doing so.

To assess intervention engagement and adherence we collected data on participant’s activity within the online intervention, including total time spent on the intervention and number of modules accessed. Completion of the intervention was determined by accessing >10 of the 14 modules. Participants who completed <10 were recorded as dropouts.

Data were collected on several mental health outcomes as detailed below, reporting estimated effect sizes and variability for each outcome. The estimates along with current retention rates, to inform future sample size calculations.

To assess the feasibility of collecting outcome data using measures of stress, anxiety, depression, resilience and wellbeing. This was assessed as the number of participants completing pre, post and follow-up measures, and numbers of those who dropped out. We also calculated missing data across all measures and within individual measures. Acceptability of measures were assessed qualitatively through semi-structured interviews conducted post intervention (2 weeks after baseline). Interview questions focused on the relevance, duration and usefulness of the outcome measures. For example, ‘Can you tell me about your experience of completing the questionnaires?’ and ‘We assessed depression, anxiety, well-being and resilience, are these important things to assess for carers? Are there other things that we should have considered?’.

Our secondary outcome was to examine the impact on family carers’ mental health to guide a future trial. Depression, anxiety and stress were assessed using the DASS-21 [22], social connectedness using the Social Connectedness Scale Revised (SCS-R) [23], well-being using the Warwick-Edinburgh Mental Well-being Scale (WEMWBS) [24] and resilience using the Resilience Scale [25]. Measures were taken at two time points, with a follow up measure for the intervention group: before the intervention (baseline), at the end of the intervention (2 weeks following baseline) and three months after baseline. These timing were changed from the original protocol, with the research team deemed it to be an unnecessary burden to ask those in the control group to complete the 3^rd^ measure given the primary aim of this study was about feasibility not effect. In addition, the 3 month follow up measure was primarily aimed at exploring if any changes in the intervention group had been sustained and was not aimed at measuring effects between the control and intervention group. Measures were not always completed within these time frames, largely due to participants in the intervention taking longer than the two weeks initially given. Average duration of completion of the intervention and range are provided below.

The WEMWBS was scored by summing the scores for each of the 14 items, which are scored from 1 to 5. Scores range from 14 to 70 with higher scores indicating more positive mental well-being [24]. Previous research found that a change of around 3 or more points could be considered clinically significant [26]. Each of the three DASS-21 sub-scales contains 7 items, with 21 items in total. Items are rated on a 4-point Likert scale. Scores for depression, anxiety and stress were calculated by summing the scores for the relevant items and multiplying by 2, with potential scores ranging from 0 to a maximum of 42, as scoring is based on the full 42-item version [22]. Higher scores indicate higher levels of depression, anxiety and stress. For resilience, items are rated on a 7-point Likert scale. The responses are summed (minimum score of 25 to a maximum of 175) with higher score reflecting stronger resilience [25]. Social connectedness responses are rated on a Likert scale from 1 ‘strongly disagree’ to 6 ‘strongly agree’. Negatively worded items are reverse scored and summed together with the positively worded items to create a score ranging from 20 to 120. Higher scores on the SCS-R reflect a stronger sense of social connectedness [23].

### 2.8. Sample size

As this was a pilot study, a sample size calculation was not performed. We aimed for 120 family carers based on previous studies and best practice [27–29]. These numbers were deemed sufficient to test and inform the practicalities of delivering an online intervention including recruitment, uptake, and attrition [17–19].

### 2.9. Random allocation

Random allocation to the intervention and control conditions was undertaken by an independent third party not involved with the research (an independent academic who was not involved in the research). Participants were randomly allocated to the intervention or wait-list control group, using a ratio of 1:1. Random allocation was computer generated, using the Random.org (https://www.random.org/lists/) online service which uses atmospheric noise to ensure randomness. Once consent was obtained to take part in study, participants we allocated either intervention or control based on the random allocation number sequence. Once those in the intervention group had completed their baseline measures, the research team provided them with access details (username and password) and instructions on how to navigate the online intervention. The control group were informed that they would receive access to the online intervention two weeks later.

### 2.10. Blinding

The research team were blind to allocation. Participants completed all psychometric measures online with no data collected by members of the research team. Thus, the research team were blind to outcome measurement. Due to the difference in timings of the intervention (i.e., wait list control), it was not possible to blind participants to group allocation.

### 2.11. Harms monitoring

Potential harms and adverse events were monitored throughout the study. Participants were advised to report any distress or negative effects associated with study participation directly to the research team contact (RL). Additionally, validated measures of depression, anxiety, and stress were reviewed to identify any clinically meaningful deterioration. If a carer scored in the severe or extremely severe range in the DASS-21. A letter was sent encouraging the carer to seek support from their GP and signpost them to other support services that they can access. If carers became distressed during the process evaluation interviews or peer support sessions, the interview/session was paused and the researcher checked spoke to the carers individually. If they were willing to continue after a short break the interview/session resumed. If they preferred to end their participation they were free to do so. The following day, the researcher telephoned the participant to check how they are feeling. If they were still upset, they were encouraged to contact their GP for additional support.

### 2.12. Analysis

**Fig 1:**
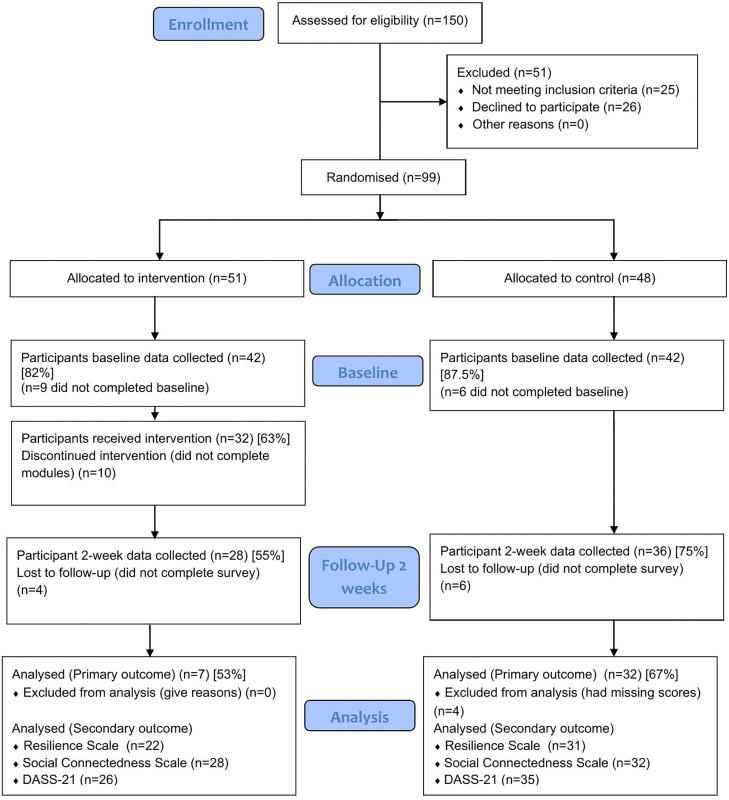
Flow of participants through the trial.

Descriptive statistics were undertaken to report quantitative data, with inferential statistics utilised to explore group differences on the outcomes measures. A CONSORT flow diagram (Fig 1) was used to describe the number of participants who were identified, recruited, commenced and finished the intervention (objective 1), with recruitment and retention rates reported as a percentage. Reasons for drop out were reported narratively. For intervention engagement and adherence (objective 2), descriptive statistics, such as percentage of those who completed the intervention and average duration are reported. These are supplemented with qualitative results from process evaluation interviews (described below). Descriptive statistics (number and percentage) were used to report number of participants completing pre, post and follow-up measures, and numbers of those who dropped out. Missing data is also reported descriptively across all measures and within individual measures (objective 4). Qualitative data on the feasibility of outcomes measure is reported narratively (objective 4).

Descriptive statistics were undertaken to present baseline characteristics. All participants were analysed in the groups to which they were randomised. However, we only included participants with complete data for each measure. Given the feasibility focus of the study, analyses were conducted using a complete-case approach. Complete-case analysis allows for a clear description of observed feasibility outcomes and patterns of missingness without introducing additional assumptions through imputation. Missing data were not imputed, and analyses were restricted to participants with available outcome data at each time point. For each outcome measure the mean difference alongside 95% confidence intervals are reported (objective 5). Inferential statistics (analysis of covariance — ANCOVA) were used as indicators of difference between the intervention and control arms, adjusting for baseline scores (objective 5). Prior to analysis, normality was assessed through the Shapiro-Wilk test of normality and Q plots. Levene’s test was used to test for homogeneity of variance. Visual inspection of the data, along with box plots and histograms were used to check for outliers. IBM SPSS Statistics 27 software was used for all analyses [30].

Qualitative data including interviews with intervention participants, comments and feedback from participants who dropped out, were analysed through reflexive thematic analysis [31] by identifying themes around perceived impact, relevance, and suggestions for improvements. Interviews were reported and transcribed verbatim. Qualitative and quantitative results were integrated where possible to provide a deeper understanding of study objectives.

## 3. Results

### 3.1. Participant recruitment and retention

One hundred and fifty family carers expressed interest in participating and were screened. Of these, 99 met the eligibility criteria (reasons for exclusion were declined to participate and did not meet inclusion criteria), consented to take part and were randomly allocated (intervention = 51, control = 48), with 84 completing baseline measures.

Recruitment began in May 2023 and completed in January 2025. By December 2023 70% of the total participants had been recruited, with the remaining 30% recruited between January 2024 to January 2025. As discussed above, we trialled two recruitment strategies, firstly via charities, and then via NHS ID teams. Rates of recruitment via charities were less than expected, therefore, in June 2024 we began recruitment via NHS ID teams in England, Scotland and Wales. The two recruitment methods ran concurrently from June 2024 to January 2025 therefore it was not possible to determine where the remaining participants were recruited from (charities vs NHS).

Of the 99 participants who were randomised, 64 were retained at two weeks post-randomisation and were included in the final analysis, equating to a 65% retention rate. At two weeks post-randomisation, 55% (intervention n = 28) and 75% (control n = 36) of participants completed follow-up measures. For the intervention group, 21 participants were retained at 3 months post-randomisation, equating to a 41% retention rate. Reasons for drop out were difficult to determine, as many carers did not provide these. However, for those who did, the most common reason for drop out was due to a lack of time due to the high care burden and complexities of the carers life.

Full details of recruitment and retention are reported in the CONSORT diagram (Fig 1).

Demographics are provided for the 84 participants who completed the baseline measures. In total there were 12 males and 72 females enrolled in the study, with a mean age of 54 years (SD = 10.1, min = 28 and max = 87 years). In terms of relationship to the person they cared for, 61 participants were mothers, 9 were fathers, 7 were siblings, and 7 were classified as “other”. Average length of time caring was 19.6 years (SD = 11.94) ranging from 1 to 50 years. On average, participants had 3 (SD = 1.3) people in their household, ranging from 1 to 7 people. Four countries and regions of the Untied Kingdom were represented, with 32 from Northern Ireland, 23 from England, 19 Scotland, and 10 from Wales. No significant differences were observed between intervention and control groups; demographics of participants by trial arm are reported in Table 2.

**Table 2:** Demographics of participants by trial arm.

Demographics	Intervention (n = 42)	Control (n = 42)
**Gender** N (%)		
Male	5 (12%)	7 (17%)
Female	37 (88%)	35 (83%)
Age (Mean (SD) (min-max)	53 (9.4) (28-78)	54 (10.1) (34-87)
**Relationship to person caring for** N (%)		
Mother	30 (71%)	31 (74%)
Father	4 (10%)	5 (12%)
Sibling	2 (5%)	5 (12%)
Other	6 (14%)	1 (2%)
**Length of time in caring role (years)** Mean (SD) (min-max)	19 (12.03) (1-50)	20 (11.99) (4-50)
**Region** N (%)		
England	10 (24%)	13 (31%)
Scotland	13 (31%)	6 (14%)
Wales	5 (12%)	5 (12%)
Northern Ireland	14 (33%)	18 (43%)
**Number in household** (Mean (SD) (min-max)	3.5 (1.5) (1–7)	3.3 (1.1) (1–6)

Comparison of those who dropped out 2 weeks post randomisation and those who completed the study, showed no statistically significant differences across baseline measures (Table 3).

**Table 3:** Comparison of demographics and outcomes measures with participants who dropped out.

	Dropped out (N = 20)	Completed study (N = 64)	P value
**Gender** N			
Male	2	10	
Female	18	54	
**Age** (Mean (SD) (min-max)			
**Length of time in caring role (years)** Mean (SD)	22 (13.43)	19 (11.47)	
**Region** N			
England	7	16	
Scotland	5	14	
Wales	0	10	
Northern Ireland	8	24	
**DASS-21: Stress Mean (SD)**	18.89 (6.83)	18.90 (5.42)	0.99
**DASS-21: Anxiety Mean (SD)**	14.22 (5.25)	13.73 (5.40)	0.73
**DASS-21: Depression Mean (SD)**	16.05 (4.81)	15.90 (5.84)	0.92
**WEMWBS**	41.37 (10.17)	38.92 (9.39)	0.33
**Resilience**	117.12 (25.41)	120.90 (23.94)	0.57
**Social Connectedness**	70.25 (20.39)	73.03 (20.19)	0.62

*Significance level <0.05.

#### 3.1.1. Process evaluation participants and data.

In addition to the main sample for the trial, a subset of participants (n = 5) consented to take part in a process evaluation interview. All participants were female and were mothers of the person they cared for. Ages ranged from 48 to 60 years old, with length of years caring ranging from 9 to 27 years (mean = 21 years). Two participants were from Scotland, two were from Wales with one from England. Process evaluation data focused on themes around perceived impact, relevance, and suggestions for improvements. Results are integrated with quantitative data within the sections below to provide a deeper understanding of study objectives.

### 3.2. Harms observed

No study-related adverse events or harms were reported during the intervention or follow-up period.

### 3.3. Adherence and engagement to the Carers-ID intervention

Out of the 42 participants in the intervention group provided with log in details to access the intervention, the majority adhered to and the completed the intervention (n = 32, 76%), defined as completion of 10 or more of the 14 modules within the programme. Reasons for non-adherence were difficult to determine, as many carers did not provide these. For those that did (n = 4), not having the time to complete and needing to prioritise own health needs were the main contributing factors to non-adherence. As detailed previously, participants were given 2 weeks to complete the 14 modules, however, they could complete the modules intermittently and at times that suited them. The majority of participants (n = 20/28) did not complete the intervention with the intended timeframe of two weeks, with time ranging from 14 days to 47 days (mean 24 days (SD 9.48).

Process evaluation interviews added to the quantitative findings, emphasising the ease of use and utility of the online intervention. Interviews highlighted that participants appreciated the online, self-directed nature of the intervention. They commented on how they could access the programme at times that suited them, and could leave it and go back, as required. As one participant commented: *“I think I have to dip in and out as well because the fact of life that gets in the way and stuff so it’s really hard to do it all at once. It’s nice that you can just go in and have a look.....the fact you can go back to it whenever you want and things like that as well is really useful.”* For carers, having information was essential for them to manage their caring role. The Carers-ID intervention provided that source of information on a board range of topics, written from a personal perspective which participants valued: *“I think knowledge is power, so any information is useful”.*

One of the main challenges within this feasibility trial was the retention of participants. As described previously, those who dropped out of the intervention were invited to take part in a follow up interview or could email feedback regarding drop out. Overwhelmingly time was seen as the biggest factor leading to non-completion of the intervention. This was confirmed through process evaluation. Participants talked about the uncertainty of the caring role, with potential crises or issues arising unexpectedly which impact on their capacity to engage in such an intervention:

*“I mean, you know, being a carer you never know what’s coming with the fun and devastation of being a carer. I don’t know what’s coming in the next hour. I could have a telephone call and now one of my children is in crisis. So yeah, you have to visit it [Carers-ID website] when you’ve got the time, and you know things are pretty stable at that time.”* However, despite these challenges participants spoke about the merits of setting aside time to engage in something that was solely for them: *“sometimes, and this is going to sound really silly, but sometimes actually having an hour to put aside every few weeks [for the peer mentoring] is good because it changes the dynamics of your day even just for an hour*.”

#### 3.3.1. Peer mentoring engagement.

Peer mentoring sessions ran on a monthly basis from July 2023 to January 2025 (15 sessions in total). A core group of 4 family carer facilitators led sessions, with leadership rotating on a monthly basis. Prior to commencement, peer mentors received training and guidelines were developed and discussed to establish boundaries. Sessions ran during working hours (9am to 5 pm), through agreement of attendees, and were 1 hour in duration. Across the 15 sessions delivered, 21 participants attended, with numbers ranging from three to 10 participants per session. Participants’ attendance ranged from one session to all 15 sessions.

Feedback through process evaluation of peer mentoring was overwhelmingly positive. Four out of the five participants had taken part in the peer mentoring sessions and provided qualitative feedback. The feeling of loneliness experienced by all participants emerged as a theme in interviews and the importance of knowing that others were facing similar issues. Participants acknowledged the value of sharing lived experiences and felt that hearing from other carers had the effect of validating their own concerns. Peer support in Carers-ID played a significant role in reassuring those who participated: *“It can be really dark and lonely and really not a nice place to be a carer sometimes and I think just somebody else saying, ‘I went through that and look at me now’, it’s just helpful when you’re in the middle of that darkness”.* This shared sense of loneliness helped to validate how participants were feeling, seemingly having a beneficial impact: *“This could make me feel better and could be the first time they have ever spoken with another carer. Is this strange thing, isn’t it? Knowing that someone else is feeling like you makes you feel better and you are not on your own”.*

Another theme to emerge in relation to peer mentoring was that of hope. Participants indicated that it was hopeful to hear what other people had gone through and how they had coped. Hearing from other carers allowed participants to reflect on their own situation and take some comfort that others had gone through similarly challenging experiences: *“It gave me a wee bit more positivity (… …) It gave me a wee bit of a spring in my step for the rest of the day and I’m also taking on other people’s stories and their situations and I suppose I’m kind of a people person. So yeah, it gave me other stuff to think about. So yes, it was positive. I did feel a bit more hopeful after them.”*

### 3.4. Feasibility of collecting outcome data

Of the participant-reported outcome measures returned, a small number were unusable due to incomplete data, meaning that these could not be analysed for those participants. This included the WEMWBS (baseline n = 2, 2 weeks n = 3), DASS-21 (baseline n = 5, 2 weeks n = 2), Social Connectedness Scale Revised (baseline n = 1, 2 weeks n = 3), Resilience Scale (baseline n = 3, 2 weeks n = 8). In addition, at baseline a further 5 participants returned the questionnaire with missing data across all measures, and 1 participant at 2 weeks’ post-randomisation. These could not be analysed for those participants. The measure with the most missing data was the Resilience Scale (n = 8) at 2 weeks post-randomisation. Of note, the Resilience Scale is the longest of the 4 measures, with 25 items.

Participants’ qualitative data showed that the measures were straightforward and unintrusive. Family carers commented on how they could see why they were being asked the questions: *“they were relatively straightforward I think, and I appreciate that they’re necessary for projects and for research like yourself, you have to evaluate”.*

Participants also commented that they appreciated being part of the research and felt the measures provided them the opportunity to consider how they were feeling. Participants valued the time to reflect on their well-being and mental health: *“they’re getting you to think about how you’re feeling again, and you do need constant reminding to think of yourself in all this”.*

### 3.5. Exploratory analysis of participant outcomes

Summary statistics for each outcome measure, for intervention and control groups, are presented in Table 4. A further breakdown of summary statistics for each outcome measure across groups and time points is also available in the supporting information (S1 Appendix). Well-being increased for the intervention group at both two week (mean = 40.11 to 40.93) and three month time points (mean = 41.05). For the control group well-being decreased at two week post-randomisation. Resilience increased for the intervention group two weeks post randomisation (mean = 125.26 to 128.77), however decreased at three months post-randomisation (mean = 123.82). For the control group resilience stayed the same across time points. Social connectedness decreased across all time points in both the intervention and control groups. Depression and stress both decreased for the intervention at both two weeks and three months post randomisation. Anxiety, increased slightly for the intervention group at two weeks (mean = 13.11 to 13.32), however decreased at three months post randomisation (mean = 11.61). Depression, stress and anxiety decreased at two weeks post randomisations for the control group.

**Table 4:** Summary statistics – Mean (SD) for outcomes measures across group.

Variable	Intervention Mean (SD)			Control Mean (SD)	
	Baseline	T2	T3	Baseline	T2
**Well-being**	40.11 (9.80)	40.93 (10.05)↑	41.05 (12.63)↑	38 (9.10)	37.64 (11.02)↓
**Resilience**	125.26 (22.32)	128.77 (18.59)↑	123.82 (20.87)↓	117.48 (24.93)	117.48 (21.17)→
**Social Connectedness**	73.89 (20.88)	72.36 (21.18)↓	69.87 (22.94)↓	72.34 (19.91)	68.51 (19.46)↓
**Depression**	15.85 (6.44)	15.33 (5.27)↓	13.06 (5.64)↓	15.94 (5.42)	14.25 (5.36)↓
**Stress**	18.86 (5.54)	16.11 (4.83)↓	15.89 (4.73)↓	18.94 (5.39)	15.63 (4.83)↓
**Anxiety**	13.11 (4.98)	13.32 (5.26)↑	11.61 (4.95)↓	14.19 (5.72)	12.28 (4.93)↓

Results from the analysis of covariance comparing differences between the intervention and control groups, adjusting for baseline scores, for each outcome measure are presented in Table 5.

**Table 5:** Analysis of covariance: Intervention vs control 2 weeks post-randomisation.

Measure	*Adjusted mean difference (SE)	N (intervention, control)	P Value	95% CI
**Well-being**	3.42 (2.84)	27,32	0.23	−2.26, 9.11
**Resilience**	12.20 (5.83)	21,31	0.04	0.48, 23.92
**Social Connectedness**	5.09 (5.12)	28, 32	0.32	−5.17, 15.35
**Depression**	0.98 (1.40)	26, 35	0.49	−1.82, 3.79
**Stress**	0.42 (1.26)	27, 34	0.74	−2.10,2.95
**Anxiety**	1.06 (1.32)	27, 36	0.43	−1.58, 3.70

*Adjusted for baseline scores. Mean difference is calculated for intervention vs control.

#### 3.5.1. Well-being outcome.

Two-week post-randomisation well-being scores between groups (intervention and control) were compared using a One-Way Between-Subjects ANCOVA, controlling for baseline scores. Results indicated there was a non-significant effect for well-being, F (1,56) = 1.45, p = 0.23, ηp2 = 0.025. On average, the intervention group (M = 40.86, SE = 2.08) scored higher than the control group (M = 37.43, SE = 1.91).

#### 3.5.2. Resilience outcome.

Two-week post-randomisation resilience scores between groups were compared using a One-Way Between-Subjects ANCOVA, controlling for baseline scores. Results indicated there was a statistically significant effect for resilience scores, F (1, 49) = 4.37, p = 0.04, ηp2 = 0.082.

On average, the intervention group (M = 129.04, SE = 4.49) scored higher than the control group (M = 116.84, SE = 3.69). Post hoc comparisons were conducted using the Bonferroni correction. The difference between intervention and control groups, 12.20, 95% CI [0.48, 23.92], was statistically significant (p = 0.04).

#### 3.5.3. Social connectedness outcome.

One-Way Between-Subjects ANCOVA for the two-week post-randomisation social connectedness scores, controlling for baseline scores, indicated a non-significant effect for social connectedness, F (1, 57) = 0.99, p = 0.32, ηp2 = 0.02.

#### 3.5.4. Depression, anxiety and stress outcomes.

Two-week post-randomisation scores between groups for depression anxiety and stress showed no statistically significant differences (Depression F (1, 58) = 0.49, p 0.49, ηp2 = 0.01), (Anxiety, F (1,60) = 0.64, p 0.43, ηp2 = 0.01), (Stress, F (1, 48) = 0.11, p 0.74, ηp2 = 0.00).

## 4. Discussion

The primary aim of this pilot study was to assess the feasibility of delivering an online intervention to family carers of individuals with ID. The study demonstrates preliminary indications of feasibility and acceptability of online delivery; however, given the major limitations outlined above, these findings should be regarded as exploratory.. When considering the pre-defined criteria for determining progression to a largescale effectiveness trial, two out of four criteria were met. The trial successfully recruited and enrolled 99 participants, meeting the > 90 participants criteria and indicting feasibility of recruitment methods. In addition, outcome measures were judged as acceptability to participants, being described as unintrusive and providing opportunity to reflect. Criteria of retention and completion of measures by >80% of participants were not met. Retention at two weeks post randomisation was 65%, signifying the need to adjust sample size calculations accordingly in future trials. While outcome measures were not completed by >80% of participants, there was minimal missing data across all time points and measures, demonstrating some level of feasibility. Other findings suggest that intervention adherence and engagement was positive. Furthermore, exploratory analyses indicated positive trends in well-being and reductions in depression, anxiety, and stress at both post-intervention and three-month follow-up for the intervention group.

### 4.1. Recruitment and strategies

The trial successfully enrolled 99 participants via two distinct recruitment methods, thereby meeting our initial feasibility criterion for progression to a definitive trial. This is a notable strength, particularly when compared to previous feasibility trials with family carers. For example, Borek et al. [17] struggled to recruit beyond a small sample of seven parent carers. Flynn et al., [19] and Coulman et al., [18] recruited 60 and 74 family carers respectively. The current feasibility trial tested two recruitment methods; via charitable organisations and NHS ID teams. As both methods ran concurrently, it was not possible to compare strategies. However, examining recruitment rates over the course of the trial, after 8 months, 70% of the participants were recruited via charitable organisations. The remaining 30% were recruited over the following 12 months via both charities and NHS services. Other comparable studies have utilised recruitment methods such as online advertisement [17,19] and organisations that offered services to carers [18]. Based on our findings, it is feasible to recruit family carers, however it is unclear how effective NHS focused recruitment strategies are.

### 4.2. Retention challenges

Participant retention posed a challenge within the current feasibility trial. Sixty-four participants (65%) were retained at the two-week follow-up, falling short of our retention target. With notable differences between intervention and control groups (55% vs 75% respectively completing 2-week measures). While this feasibility criterion was not met, retention rate aligns with other comparable trials [18,19]. Flynn et al., [19] reported 70% retention at 6 months post-randomisation, while Coulman et al., [18] had 72% retention at 12 months. Recruitment of family carers for dementia [32], foster carers [33] and adults with chronic conditions [34] have reported similar rates. These comparable retention rates indicate the difficulty of maintaining engagement with carers, whose time is constrained by the complex and unpredictable demands of caregiving. Feedback from the process evaluation interviews echoed this, highlighting time scarcity as a barrier. Studies have found that building relationships, aligning values, and establishing trust are key strategies for effective recruitment [35]. There is a need to anticipate specific attrition challenges for family carers in future trials and adjust sample size calculations accordingly. Based on the 65% retention rate, future definitive trials should account for a 35–40% attrition rate and use this to calculate the required sample size. In addition, future trials should systematically record reasons for non-compliance where possible to help inform further studies.

### 4.3. Outcome measure acceptability and feasibility

Outcome measures proved acceptable to participants, with minimal missing data. The use of a well-being measure as the primary outcome aligns with other studies among family carers of people with ID [17–19], and carers of individual with other conditions [36–38] reinforcing its relevance and suitability in this population. However, Coulman et al. [18] in their feasibility trial of carers of children with ID found that lengthy measures may impact completion rates; thus, optimisation and streamlining of questionnaires should be considered in future designs.

While exploratory analyses suggest potential benefits of the intervention, primarily in well-being, depression, anxiety, and stress. The results are preliminary and not sufficient to support definitive conclusions due to the study’s limited power and methodological constraints. Further trials should also include the control group in the follow up measurement to determine differences between groups. Although preliminary, these results warrant further investigation in a larger, adequately powered trial.

### 4.4. Adherence and intervention completion

Participant adherence to the intervention was encouraging, with most completing the modules as expected. Out of the 42 participants who started the intervention, 28 completed, defined as completion of 10 or more of the 14 modules within the programme. As above, there was a notable difference in drop out between the intervention and control group. This raises questions about the feasibility of delivering the Carer-ID intervention utilising the current methodology. One area of consideration is that participants (n = 20/28) did not complete the intervention with the intended timeframe of 2 weeks, with time ranging from 14 days to 47 days (mean 24 days (SD 9.48). Previous studies among carers of people with ID also encountered challenges with intervention completion. Flynn et al. [19] noted that only 3 out of 27 family carers completed the intervention within a 4-week timeframe, with a mean completion time of 65 days. Despite these challenges, qualitative data suggests that carers accessed the content flexibly, dipping in and out based on their availability. This trend suggests that adaptive delivery models—with flexible timelines and self-paced content—may be more effective at increasing completion rates. Findings reinforce the importance of accommodating variability in carer schedules and to consider appropriate timeframes to allow carers to fully complete interventions. Thus, future studies should consider this in trial design among this population.

### 4.5. Strengths and limitations

One of the primary strengths of this trial was its large and geographically diverse sample, recruited from across the UK. Additionally, the inclusion of an embedded qualitative component (process evaluation) allowed for a more in-depth examination of key feasibility criteria. Recruitment strategies were refined iteratively, enabling the evaluation of multiple recruitment pathways.

However, the study also had several limitations. The short retention window and relatively high dropout rate may limit the generalisability of the findings. The sample was predominantly female, with comparatively fewer male participants, which may affect the applicability of results across genders. In addition, participants were not blinded to allocation and self-reported on outcomes measures. Moreover, the inability to gather feedback from participants who disengaged from the study limited our understanding of the barriers to retention. A smaller than anticipated sample of carers contributed to the process evaluation (5 out of a target of 12). It is possible that this sample was highly selective and thus findings should be interpreted cautiously.

Baseline differences in well-being and resilience were observed between the intervention and control groups, alongside higher attrition in the control group. To address these imbalances, all analyses were adjusted for baseline levels of the outcome variables, and models capable of handling missing data were employed. Nonetheless, the presence of differential dropout suggests that selective attrition cannot be entirely excluded. As such, the results should be interpreted cautiously, and future studies should aim to minimise attrition and ensure better baseline equivalence.

## 5. Conclusions

This pilot study demonstrated preliminary indications of feasibility to recruit to the Carer-ID intervention. However, significant challenges and limitations were present in this current study. Retention remains a key challenge, likely influenced by the demands placed on carers. While the intervention showed promise, particularly in terms of adherence and initial outcome trends, a definitive trial would require tailored strategies to improve retention and optimise intervention delivery. Future trials should incorporate strategies to enhance retention, such as reminder systems, incentives, or shorter and more engaging outcome measures. Sample size calculations should also account for anticipated attrition specifically for this population. Additionally, allowing greater flexibility and length in intervention timelines could better reflect the real-life constraints faced by carers. An adaptive, flexible model of delivery, which allows for flexible or longer timelines and self-paced content, should be considered in future trial design within this population. Further investigation of potential benefits of the Carers-ID online intervention is warranted in a larger, adequately powered trial.

## Supporting information

S1 Appendix Summary statistics for each outcome measure. (DOCX)

S2 File CONSORT checklist. (DOCX)
