# Supplementary material for: A randomised controlled feasibility study of the Carers-ID intervention to support the mental health of family carers of people with intellectual disabilities
Source: PLoS One. 2026 Mar 20;21(3):e0345096. doi: 10.1371/journal.pone.0345096 (PMC13004343; doi:10.1371/journal.pone.0345096)
Supplement: S1 Appendix — (DOCX) [file pone.0345096.s001.docx]

**S1 Appendix**

Summary statistics for each outcome measure across group and time points.

**Wellbeing**

| **Group** | **Mean (SD)** | **N** | **Min – Max** |
| --- | --- | --- | --- |
| **Baseline** | | | |
| Intervention | 40.11 (9.80) | 27 | 25 – 67 |
| Control | 38 (9.10) | 35 | 23 – 58 |
| **T1** | | | |
| Intervention | 40.93 (10.05) | 28 | 21 - 68 |
| Control | 37.64 (11.02) | 33 | 18 – 70 |
| **T2** | | | |
| Intervention | 41.05 (12.63) | 18 | 14 - 63 |

**Resilience**

| **Group** | **Mean (SD)** | **N** | **Min – Max** |
| --- | --- | --- | --- |
| **Baseline** | | | |
| Intervention | 125.26 (22.32) | 27 | 82 - 160 |
| Control | 117.44 (24.93) | 34 | 23 – 58 |
| **T1** | | | |
| Intervention | 128.77 (18.59) | 22 | 103 - 169 |
| Control | 117.48 (21.17) | 33 | 78 - 155 |
| **T2** | | | |
| Intervention | 41.05 (12.63) | 17 | 86 - 165 |

**Social Connectedness**

| **Group** | **Mean (SD)** | **N** | **Min – Max** |
| --- | --- | --- | --- |
| **Baseline** | | | |
| Intervention | 73.89 (20.88) | 28 | 41 - 115 |
| Control | 72.34 (19.91) | 35 | 29 - 115 |
| **T1** | | | |
| Intervention | 72.36 (21.18) | 28 | 32 - 116 |
| Control | 68.51 (19.46) | 33 | 41 - 112 |
| **T2** | | | |
| Intervention | 69.87 (22.94) | 16 | 31 – 102 |

**Depression**

| **Group** | **Mean (SD)** | **N** | **Min – Max** |
| --- | --- | --- | --- |
| **Baseline** | | | |
| Intervention | 15.85 (6.44) | 27 | 8 – 30 |
| Control | 15.94 (5.42) | 35 | 8 – 31 |
| **T1** | | | |
| Intervention | 15.33 (5.27) | 27 | 7 – 28 |
| Control | 14.25 (5.36) | 36 | 7 – 28 |
| **T2** | | | |
| Intervention | 13.06 (5.64) | 17 | 7 – 23 |

**Stress**

| **Group** | **Mean (SD)** | **N** | **Min – Max** |
| --- | --- | --- | --- |
| **Baseline** | | | |
| Intervention | 18.86 (5.54) | 28 | 8 - 29 |
| Control | 18.94 (5.39) | 34 | 8 - 32 |
| **T1** | | | |
| Intervention | 16.11 (4.83) | 27 | 7 – 27 |
| Control | 15.63 (4.83) | 36 | 7 – 28 |
| **T2** | | | |
| Intervention | 15.89 (4.73) | 18 | 8 – 24 |

**Anxiety**

| **Group** | **Mean (SD)** | **N** | **Min – Max** |
| --- | --- | --- | --- |
| **Baseline** | | | |
| Intervention | 13.11 (4.98) | 27 | 8 – 25 |
| Control | 14.19 (5.72) | 36 | 8 – 28 |
| **T1** | | | |
| Intervention | 13.32 (5.26) | 28 | 7 - 26 |
| Control | 12.28 (4.93) | 36 | 7 – 27 |
| **T2** | | | |
| Intervention | 11.61 (4.95) | 18 | 7 – 24 |
